# Massive retroperitoneal tubercular abscess mimicking a leaking abdominal aortic aneurysm: a case report

**DOI:** 10.1186/1757-1626-1-236

**Published:** 2008-10-14

**Authors:** Pankaj K Singh, Amir Azam, Vinay K Singh, Juhi Tomar, Alka Mishra, Kuldeep K Singh, Sanjeev Gupta

**Affiliations:** 1 Department of Neurosurgery, Royal Hallamshire Hospital, Sheffield, S10 2JF, UK; 2 Trauma & Orthopaedics Surgery, Manchester Royal Infirmary, Manchester, UK; 3 Orthopaedics & Spinal Surgery, Luton and Dunstable Hospital NHS Trust, Luton, UK; 4 Medical Education Centre, Northern General Hospital, Sheffield, UK; 5 ST-2 Department of Accident & Emergency, Bedford General Hospital, Bedford, UK; 6 SMO-Pulse Polio Programme, World Health Organization, India; 7 Consultant Spinal & Neurosurgeon, GTB Hospital, New Delhi, India

## Abstract

In spite of being a common diagnosis in the patients of Asian origin, atypical presentations of tuberculosis may pose diagnostic challenges. We report a huge prevertebral abscess in a 30-year-old female, mimicking a leaking aortic aneurysm. The patient was managed successfully by emergency decompression and stabilization. The issues related to poor patient compliance to chemotherapy and management of atypical presentations of spinal tuberculosis are discussed here.

## Introduction

Tuberculosis is considered to be a disease of underdeveloped countries, but the incidence is rising in the western world due to increasing incidence of acquired immunodeficiency syndrome (AIDS). Spinal tuberculosis is the commonest form of skeletal tuberculosis and is frequently associated with pre or paraspinal abscess [1]. Cauda equina and paraplegia are major complications of spinal tuberculosis. We describe a rare case of massive thoracic paraspinal tubercular abscess presenting as a large pulsatile epigastric mass simulating a leaking aortic aneurysm. The patient was successfully treated by two stage stabilization and remained asymptomatic in three years follow-up.

## Case presentation

A 30-year-old housewife of Asian origin diagnosed to have pulmonary tuberculosis, was referred to spinal team as cauda equina lesion by her general practitioner. She was on antitubercular treatment (ATT) for last three months and, had history of progressive weakness in lower limbs for a week with urinary incontinence for a day. On initial examination she had stable airway, breathing and circulation. Neurological examination revealed increased tone and decreased power in both lower limbs (MRC 3/5). Sensory level was present at the level of D9. Knee and ankle reflexes were increased bilaterally with up-going planters. Anal tone and perianal sensations were decreased. On catheterization she had a residual volume of 400 ml. The clinical examination revealed a mixed picture. Whereas a sensory level at D9 with upper motor neuron signs in lower limbs (increased jerks and up-going planters) indicated a compressive lesion at D9, urinary retention along with decreased perianal sensations and, impaired anal tone and reflexes favoured a clinical diagnosis of cauda equina compression. Due to equivocal clinical signs initial impression was a multilevel disc disease with acute compression of cauda equina region. Urgent MR scan was done within 2 hours of clinical examination in order to rule out cauda equine lesion. On her way back from radiology department she dropped blood pressure and collapsed. Fluid resuscitation was started and re-examination confirmed large pulsatile mass in epigastrium with feeble femoral and dorsalis pedis pulses in both lower limbs. Lower extremities were cold on palpation; capillary refill was delayed to 3 sec with appreciable radio-femoral delay. Hypotension was resistant to fluid challenge and surgical team was contacted for advice. With the advice of vascular team a CT scan was requested. In view of her deteriorating clinical condition and low haemoglobin (8.5), decision to perform urgent laparotomy without CT scan was made for a possible leaking aortic aneurysm.

**Figure 1:**
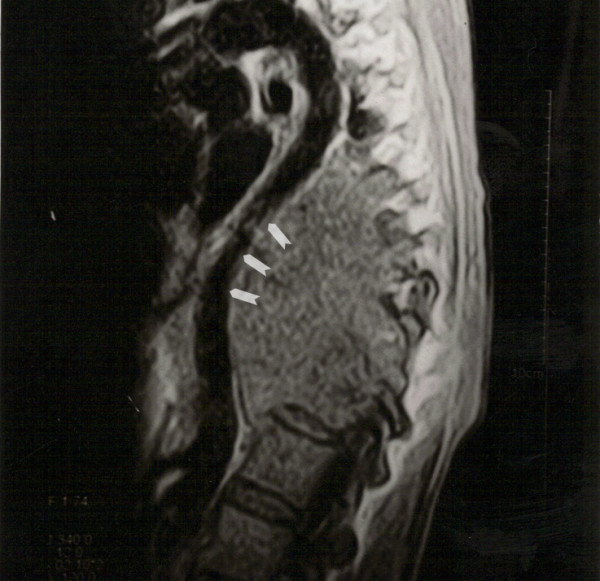
T2 weighted sagittal section showing massive prevertebral collection pushing the aorta anteriorly resulting in severe narrowing of aorta at mid thoracic level (arrowheads).

**Figure 2:**
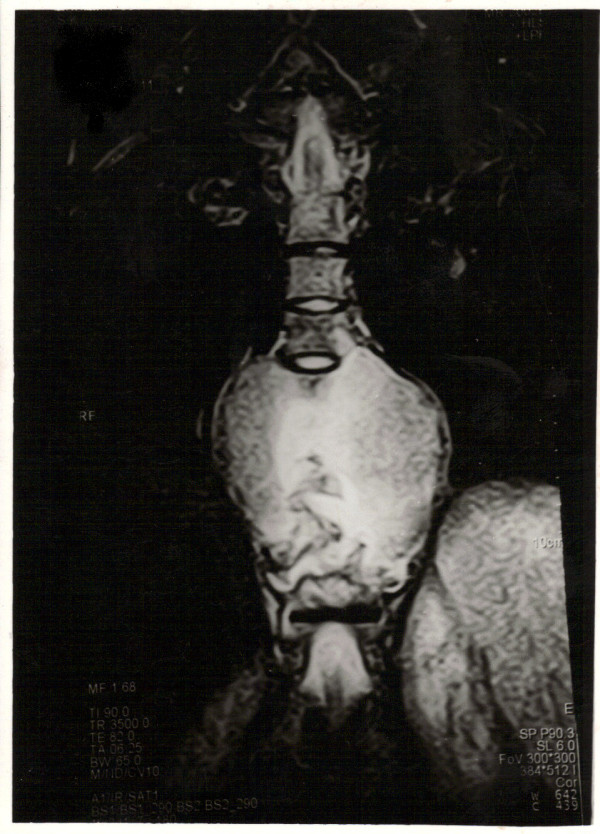
T2 weighted coronal image showing the lateral extension of abscess measuring 12 × 18 × 20 cm in maximum dimensions.

**Figure 3:**
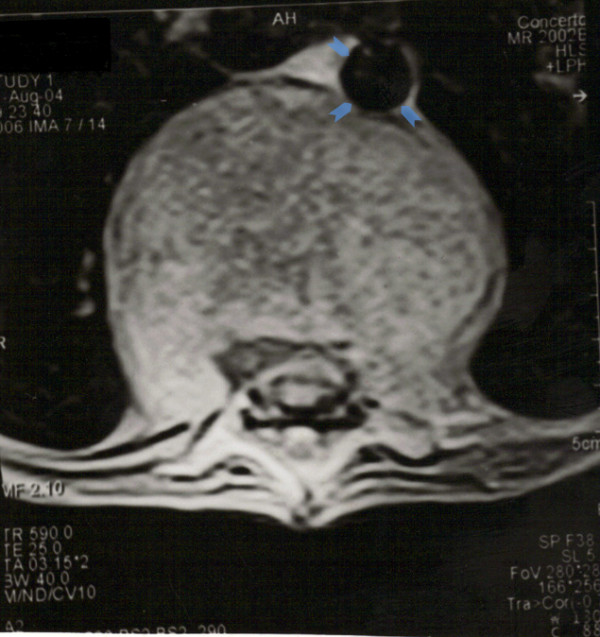
**T2 weighted axial MR sequence showing massive collection in front of spinal column extending up to aorta.** The massive size of the abscess is clearly evident as compared to size of aorta (arrowheads).

**Figure 4:**
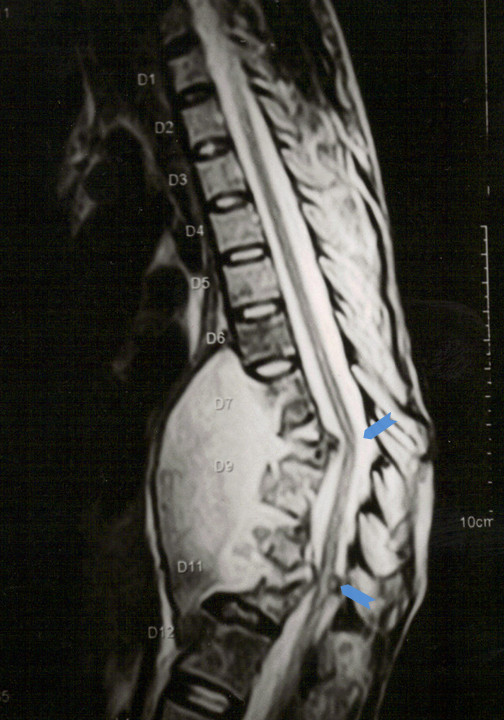
Sagittal MR image showing a huge prevertebral tubercular abscess extending from D6 to D12 levels with severe destruction vertebral bodies of D7 to D12 resulting in anterior angulation of spinal column leading to kinking of spinal cord at D7/8 and D10/11 with signal changes (arrowheads).

MRI scan was reported in the mean time which showed the presence of massive pre-vertebral abscess in the thoracic region, pushing the aorta anteriorly resulting in significant narrowing of descending thoracic aorta (Fig 1). The abscess was extending from D6 to D12 level measuring 12 × 18 × 20 cm in size (Fig 2 &3). The vertebral bodies of the D-7 to D-12 were severely destroyed, resulting in anterior angulation of thoracic spine. The spinal cord was compressed at the levels of D7/8 and D10/11 with signal changes (Fig 4).

Due to presence of the acute compression of the thoracic aorta and spinal cord an emergency surgery was performed by spinal team to relieve the compression, with vascular surgeons on standby. Spine was approached through anterior trans-thoracic route via sixth intercostal space. A big abscess compressing the aorta was encountered anterior to thoracic vertebrae extending from D6 to D-12. Aorta was severely compressed by the abscess with no evidence of pseudoaneurysm. The abscess was drained and sample was sent for microbiology. The neighbouring soft tissue was also sent for histopathological examination with excision and debridement of necrotic dead tissues. Femoral and dorsalis pedis pulses returned to normal on operating table soon after drainage of abscess. Keeping in view, the presence of a heavy infection, drains were left *in situ*, and the wound was closed without any in spinal stabilisation.

AFB staining was positive for Mycobacterium tuberculosis from pus as well as surrounding soft tissue samples, which was confirmed on culture too. Poor patient compliance to previous ATT was discovered, which was restarted again post-operatively with the advice of microbiologist. She was started on Rifampicin, Isoniazid, Pyrazinamide and Ethambutol for initial 2 months followed by Isoniazid and Rifampicin for another 10 months. The power in the both lower limbs came back to normal on second postoperative day with resolution of urinary incontinence. She responded well to the treatment with improvement in her systemic symptoms in one week. The drain was taken out on second postoperative day. The patient was re-operated after six weeks, and spine was stabilised by plating and bone-grafting. The patient was gradually mobilised and discharged home after satisfactory recovery. The patient remained asymptomatic in three year's follow-up. Pulmonary tuberculosis which flared up initially also settled after recommencing the ATT. Follow-up x-ray revealed a satisfactory spinal fusion and well aligned prosthesis *in situ.*

## Discussion

Pott's disease is an uncommon manifestation of tuberculosis, which usually involves thoracic or lumbar vertebrae. Vertebral body is most severely affected and may lead to collapse and ankylosis in later stages. A prevertebral abscess generally accompanies vertebral involvement [2]. A cold abscess as a result of tuberculosis can emerge in a number of anatomical regions, and perhaps most notably as a psoas abscess. Though large cold abscesses have been described but such a severe compression of the aorta, causing hemodynamic instability, seems to be a very rare entity and to the best of our knowledge not yet been reported in the literature.

The spinal tuberculosis is a result of haematogenous dissemination from primary focus in the lungs or the lymph nodes. The central type of vertebral tuberculosis spreads along with Batson's plexus of veins, while paradiscal infection spreads through the arteries. The anterior paravertebral abscess results from the extension of the abscess beneath the anterior longitudinal ligament and periosteum [1]. In our case probably the infection travelled from the lungs to spine.

Delayed presentation and large abscess formations occur more frequently in cases of tuberculosis infections rather than in cases of pyogenic infections. The chronic and insidious nature of tuberculous spondylitis causes late diagnosis; therefore, enough time passes for mass presentation of abscesses, which may be huge as in our case. On the other hand, frank systemic signs at an earlier stage of pyogenic abscesses allow early diagnosis and treatment, which decreases the incidence of massive abscess formation. [3,4].

Though rare but tubercular pseudoaneurysm of the aorta has been described and few cases have been reported in literature [5,6]. In our case there was no evidence of aortic pseudoaneurysm on MR scan and severe compression of the aorta was present due to mass effect of the abscess. The conventional procedure in the treatment of vertebral tuberculosis is drainage of the abscess, curettage of the devitalized vertebra and institution of ATT. Opinion varies regarding the operative indication for Pott's spine. Many surgeons perform debridement and decompression in all cases, irrespective of neurological involvement. Others perform operative decompression only in those patients who do not respond to ATT [7]. Prognosis depends on many factors; the magnitude of cord compression, duration of neural complications, age and general condition of the patient [7].

Rezai et al. reported their experience of managing 20 patients of Pott's spine. Study concluded that in selected patients, early operative treatment with instrumentation, when indicated, minimizes neurological deterioration and spinal deformity. It allows early ambulation resulting in excellent neurological outcome [7].

In a meta-analysis Turgut concluded that the neurological involvement due to Pott's disease is relatively benign if urgent decompression is performed at the onset of the disease [8]. Posterior instrumentation results are encouraging in the prevention or treatment of late kyphosis; however, a second-stage operation is needed. Benli et al reported their surgical results of 63 patients with Pott's spine who underwent anterior radical debridement with anterior fusion and instrumentation. They found that anterior instrumentation is a safe and effective method in the treatment of tuberculosis spondylitis [9]. Our patient had anterior debridement followed by anterior fusion with bone grafting and anterior instrumentation.

## Conclusion

Tuberculosis is common in patients of Asian origin and is easily diagnosed and treated. Atypical presentation can catch treating clinical off-guard and leading to incorrect diagnosis and adverse clinical outcome. Pulsatile epigastric mass, however pathognomonic of aortic aneurysm, does not hold true everytime. Poor compliance to antitubercular treatment may be a matter of concern in select group of patients. Detail history, regular follow-ups, monitoring of compliance and, patient education is vital for prompt and timely treatment of disease in this group.

## Abbreviations

ATT: Antitubercular Treatment; D: Dorsal Vertebra; AIDS: Acquired Immune Deficiency Syndrome; MRI: Magnetic Resonance Imaging; CT: Computerised Tomography; MRC: Medical Research Council grading of power.

## Competing interests

The authors declare that they have no competing interests.

## Authors' contributions

PS was the operating surgeon, collected clinical details including photographs, summarised the case history and prepared first draft. He also verified the authenticity of scientific content. AA prepared the final revision of the manuscript and gave his useful comments in discussion. VS conducted a literature search, preparation of final manuscript including grammar, punctuation and style. JT helped in final formatting, revision and submission process. She also helped in retrieving useful references through her hospital library. AM prepared the final design and formatting of final manuscript including the electronic processing of the images. SG was the Consultant Spinal & Neurosurgeon; gave his critical remarks on the manuscript, especially on the scientific content, flow, continuity and English Grammar. KS helped in final revision and formatting.

## Consent

A fully informed written consent was obtained for the publication of this case report and accompanying images. A copy of the written consent is available for review by the Editor-in-Chief of this journal.
